# Freeze-thawing impairs the motility, plasma membrane integrity and mitochondria function of boar spermatozoa through generating excessive ROS

**DOI:** 10.1186/s12917-021-02804-1

**Published:** 2021-03-22

**Authors:** Bin Zhang, Yan Wang, Caihong Wu, Shulei Qiu, Xiaolan Chen, Bingyan Cai, Huimei Xie

**Affiliations:** 1 Jiangsu Agri-animal Husbandry Vocational College, 8 Feng Huang East Road, Taizhou, Jiangsu People’s Republic of China; 2 Food, Animal and Plant Inspection and Quarantine Technical Center of Shanghai Customs, Shanghai, People’s Republic of China

**Keywords:** Freeze-thawing, Boar spermatozoa, Motility, Apoptosis, ROS

## Abstract

**Background:**

Cryopreservation is an efficient way to store spermatozoa and is closely associated with the quality of sperm after the freeze-thaw process. During freeze-thaw cycling, excessive reactive oxygen species (ROS) are produced, and the effects of ROS on boar sperm during cryopreservation have not been identified.

**Results:**

In this study, we evaluated the quality of boar spermatozoa in different steps of cryopreservation (extension, cooling, and thawing for 30 min and 240 min) with or without boar-sperm antioxidant (N-acetylcysteine (NAC)). The ROS levels, sperm motility, plasma membrane integrity, mitochondrial activity, sperm chromatin structure, ATP content, and sperm apoptosis were assayed. After thawing, the ROS level and sperm apoptosis were significantly increased, and the sperm motility, plasma membrane integrity, mitochondrial activity, sperm chromatin structure, and ATP content were significantly impaired compared with those at the extension period and cooling period. Moreover, the addition of N-acetyl L-cysteine (NAC) reversed these changes.

**Conclusion:**

The freeze-thawing of boar spermatozoa impaired their motility, plasma membrane, mitochondrial activity, sperm chromatin structure and apoptosis by producing excessive ROS. Thus, the downregulation of ROS level by antioxidants, especially the NAC, is important for manufacturing frozen pig sperm to increase reproductive cells and livestock propagation, as well as to improve the application of frozen semen in pigs worldwide.

**Supplementary Information:**

The online version contains supplementary material available at 10.1186/s12917-021-02804-1.

## Background

Artificial insemination with cryopreserved semen enables affordable, large-scale dissemination of gametes with superior genetics. Frozen sperm can supplement the limited longevity in vitro and are widely used [1]. Although physical changes during freezing and thawing can lead to changes in sperm morphology and function, affecting sperm survival and reducing fertilization ability, cryopreservation is currently the most effective method for long-term sperm preservation [2–4]. The use of frozen boar semen is limited due to the high susceptibility of boar sperm to cold shock [5, 6]. Presently, the application of frozen semen in pigs worldwide remains less than 1% [7–9]. Therefore, it is necessary to define the changes in sperm upon cryopreservation of boar semen in order to develop appropriate semen cryopreservation methods and to improve the quality of frozen semen.

Reactive oxygen species (ROS) have physiologically relevant roles in improving intracellular cAMP levels, acrosome reactions, hyperactivation and spermatozoa fusion [10–12]. Uncontrolled (i.e. excessive) ROS generation can attack unsaturated fatty acids (UFAs) on the plasma membrane, alter mitochondrial membrane potential and induce protein sulfhydryl peroxidation, thereby altering sperm function and structure [13–15], which is regarded as an important cause of sperm damage during cryopreservation [16, 17]. In addition, ROS could cause that the sperm be unable to fertilize the oocyte. Compared with other species, boar sperm are more vulnerable to peroxidative damage during cryopreservation due to its high content of polyunsaturated fatty acids [18–20] that serve as preferred substrates for ROS generation in membranes [21], resulting in limitations of the use of frozen pig sperm [19]. Little is known about the influence of ROS on the cryopreservation of boar sperm.

N-acetyl L-cysteine (NAC), a derivative of amino acid L-cysteine, has free radical scavenging activity both in vivo [22] and *vitro* [23]. Studies have been reported that daily treatment with NAC results in a significant improvement in sperm motility in comparison to placebo and improves sperm concentration and acrosome reaction while reducing ROS and oxidation of sperm DNA [24]. Additionally, Jannatifar et al. reported that the oral supplementation may improve sperm parameters and oxidative/antioxidant status in infertile males [25]. Presently, there is no report on the addition of NAC to the freezing extender of boar sperm. Therefore, this study was aimed at defining the changes of ROS levels in sperm upon cryopreservation of boar semen and investigating the effects of NAC treatment on boar sperm during cryopreservation to find efficient antioxidant preservatives such as NAC and to improve the application of frozen semen in pigs worldwide.

## Results

### Effects of cryopreservation on the boar spermatozoa ROS levels

**Fig. 1:**
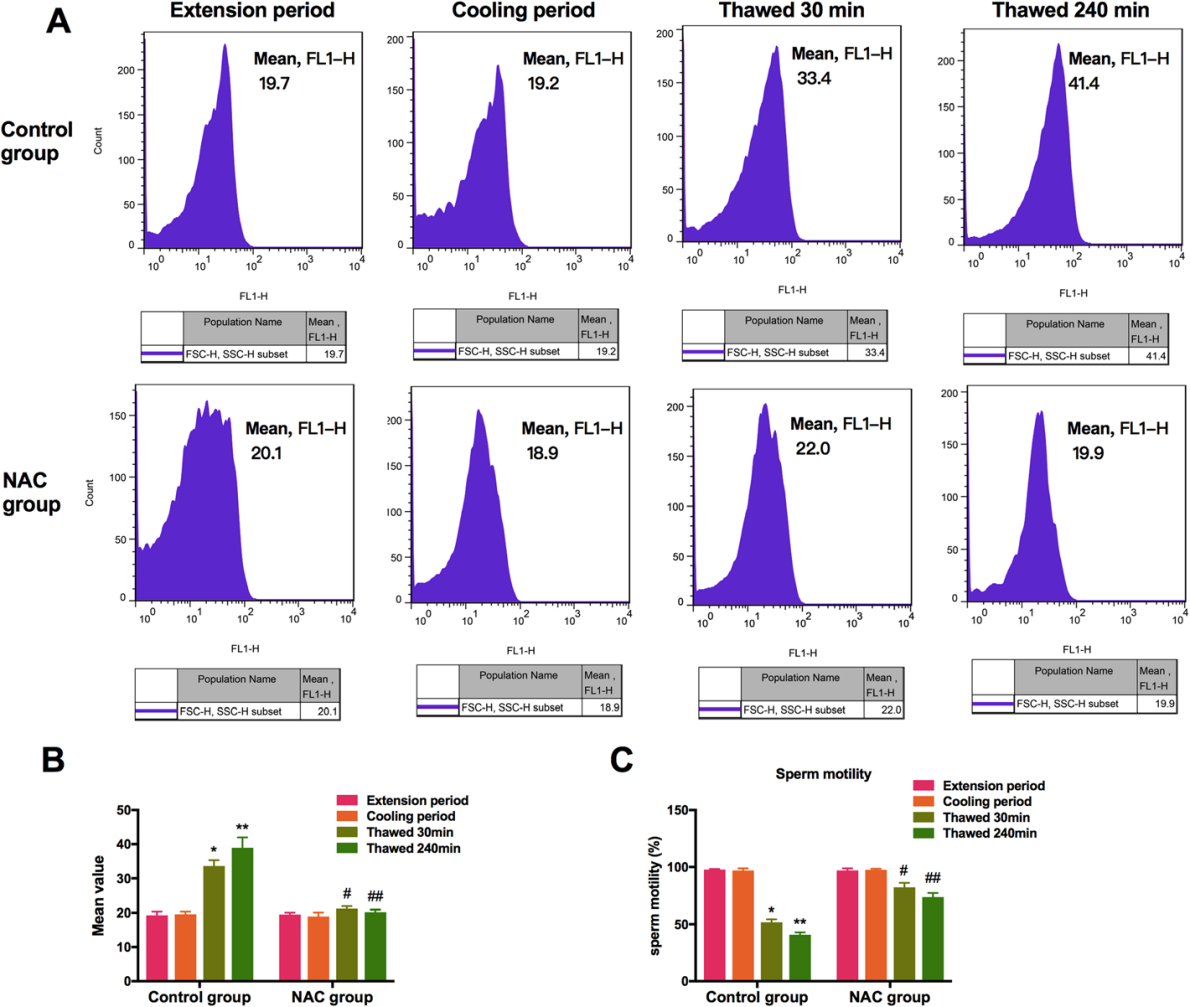
The ROS level of frozen-thawed spermatozoa with or withou NAC was detected by flow cytometry using DCFH-DA assay (**a**, **b**). The percentage of sperm motility with or withou NAC at each step of cryopreservation was determined using a computer-assisted semen analysis system (**c**). Data are presented as the means ± SE (*n* = 3). * *P* < 0.05, ** *P* < 0.01 VS the extension period of control group; # *P* < 0.05, ## *P* < 0.01 VS the thawed 30 min or thawed 240 min period of NAC group

The ROS level of frozen-thawed spermatozoa was detected by fluorescence using the DCFH-DA assay. A significantly high ROS level was observed in thawed spermatozoa with an increase in the thawing time compared with the spermatozoa at the extension period and cooling period in the control group, and the ROS level of thawed spermatozoa was inhibited in the NAC group (Fig. 1a and b).

### Effects of cryopreservation on boar spermatozoa motility

The motility of sperm is the most intuitive manifestation of frozen-thawed sperm quality. After sperm were cryopreserved for 4 weeks, the motility was assessed at the extension period, cooling period, thawing for 30 min or thawing for 240 min. The motility of thawed spermatozoa was significantly lower than that at the extension period and cooling period with thawing time in the control group. The motility of thawed spermatozoa was increased in the NAC group compared with that in the thawed groups, but the motility was still lower than that at the extension period and cooling period (Fig. 1c).

### Effects of cryopreservation on plasma membrane integrity

**Fig. 2:**
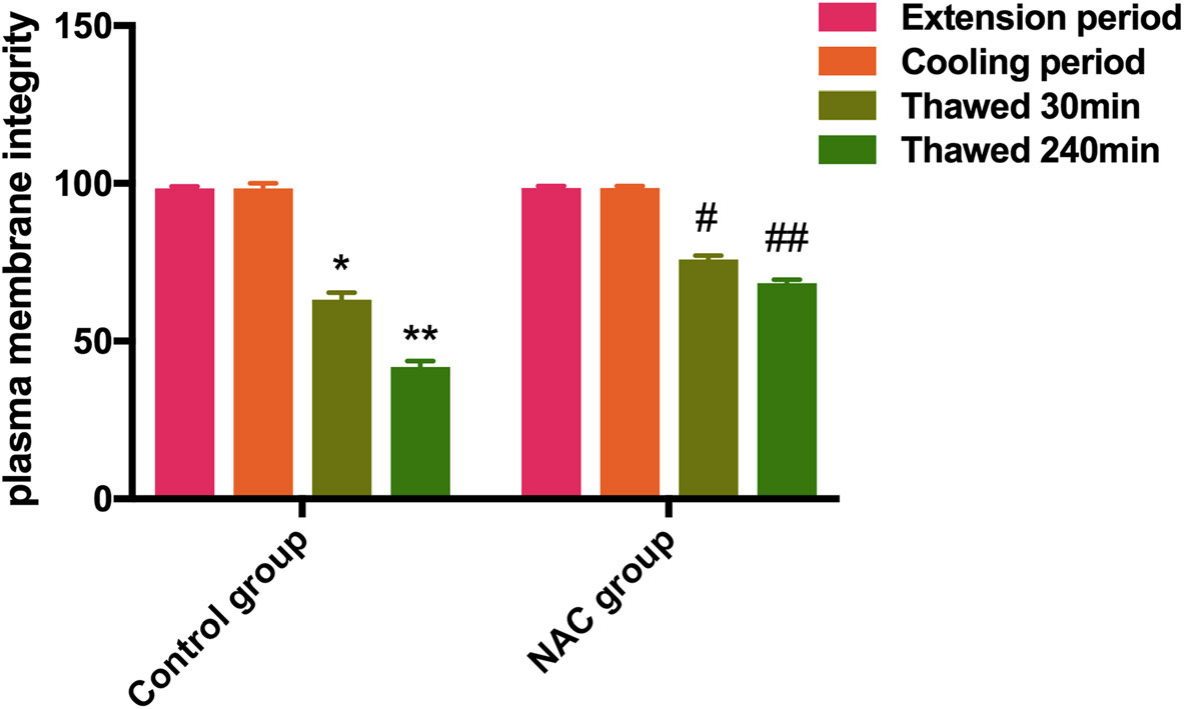
The plasma membrane integrity of the sperm was assessed by the hypo-osmotic swelling test (HOST). Data are presented as the means ± SE (*n* = 3). * *P* < 0.05, ** *P* < 0.01 VS the extension period of control group; # *P* < 0.05, ## *P* < 0.01 VS the thawed 30 min or thawed 240 min period of NAC group

The plasma membrane of sperm was completed at the extended period and cooling period. The plasma membrane integrity was significantly reduced after thawing, and the change was enhanced with increased thawing time. However, the plasma membrane integrity was significantly increased in thawed sperm with the addition of NAC (Fig. 2).

### Effects of cryopreservation on the mitochondrial function and ATP content boar spermatozoa

**Fig. 3:**
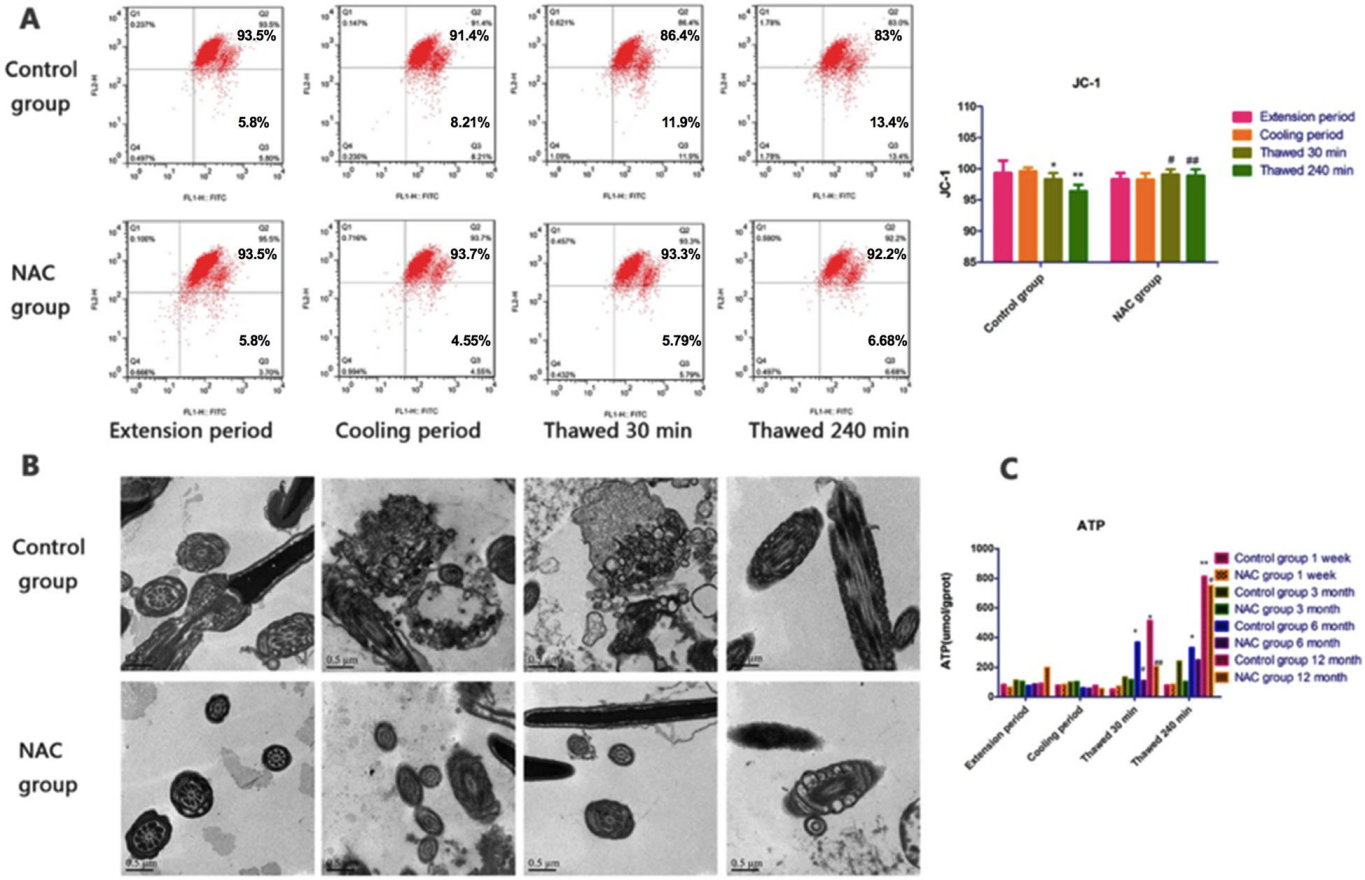
Mitochondrial function was evaluated using JC-1 (**a**), TEM ultrastructure (**b**) and ATP content (**c**). Data are presented as the means ± SE (*n* = 3). * *P* < 0.05, ** *P* < 0.01 VS the extension period of control group; # *P* < 0.05, ## *P* < 0.01 VS the thawed 30 min or thawed 240 min period of NAC group

Mitochondria are one of the only few organelles in sperm and play an important role in providing ATP for sperm movement [26]. Excessive ROS can inhibit mitochondrial function to decrease spermatozoa motility [27]. To evaluate this possibility, the mitochondrial membrane potential was evaluated by assessing the JC-1 and ATP contents using ELISA. A significant decrease in ΔΨm in thawed spermatozoa was observed compared with that in spermatozoa during the extension period and cooling period, and the effect was enhanced with increased thawing time in the control group. The ultrastructure of mitochondria had pathological changes, and the number and distribution of mitochondria were abnormal. The ATP content in thawed spermatozoa was reduced compared with that in spermatozoa during the extension period. The ΔΨm, mitochondrial ultrastructure and ATP content of thawed spermatozoa recovered to the same level as those at the extension period and cooling period in the NAC group (Fig. 3a, b, and c).

### Effects of cryopreservation on sperm DNA fragmentation

**Fig. 4:**
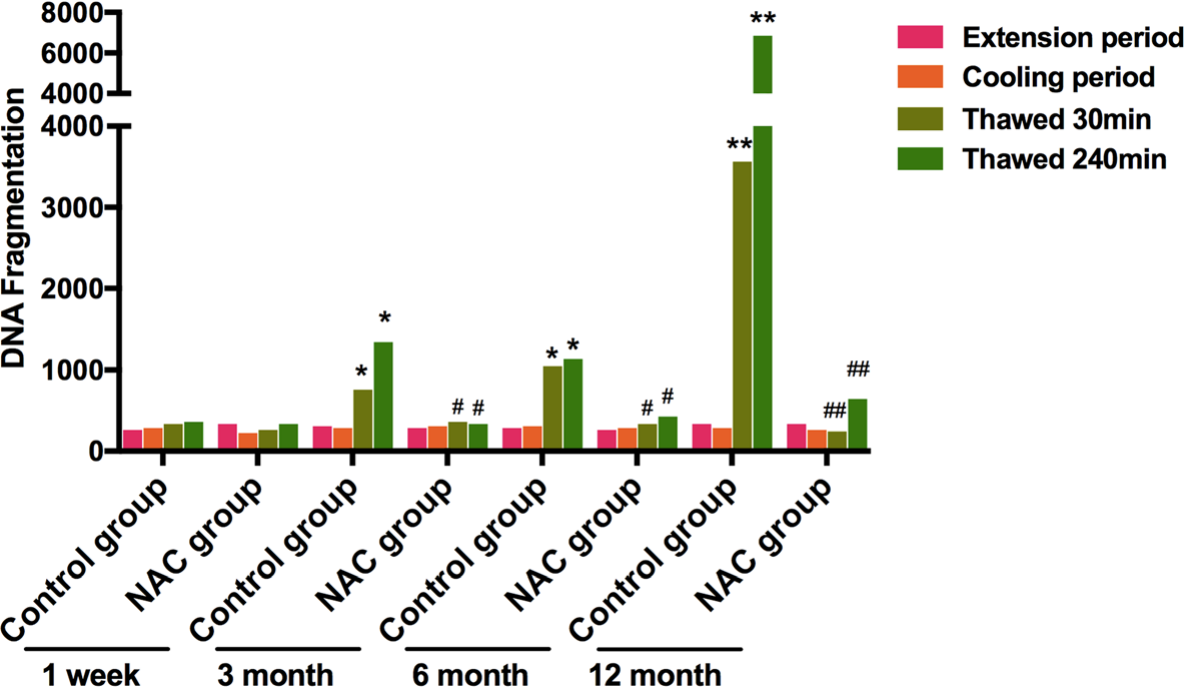
Fragmented DNA were assayed. Data are presented as the means ± SE (*n* = 3). * *P* < 0.05, ** *P* < 0.01 VS the extension period of control group; # *P* < 0.05, ## *P* < 0.01 VS the thawed 30 min or thawed 240 min period of NAC group

Few sperm with fragmented DNA were observed during the extended period and cooling period. Conversely, the percentages of sperm with fragmented DNA were significantly increased at 30 min or 240 min post-thawing. After the addition of NAC, the percentages of sperm with fragmented DNA were significantly decreased in thawed sperm (Fig. 4).

### Effects of cryopreservation on boar spermatozoa apoptosis

**Fig. 5:**
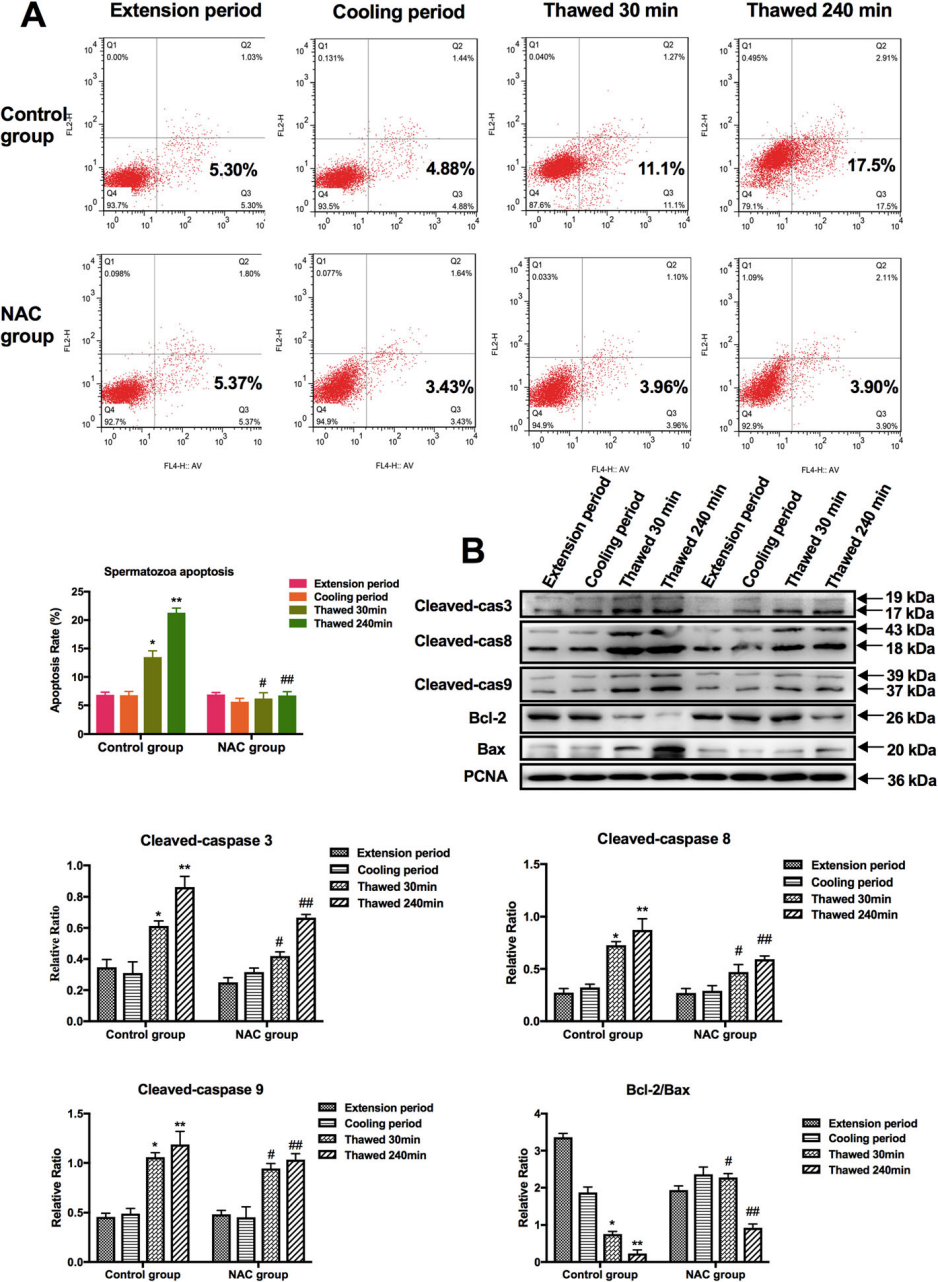
Apoptosis of sperm was detected by flow cytometry (**a**) and western blot (**b**). Data are presented as the means ± SE (*n* = 3). * *P* < 0.05, ** *P* < 0.01 VS the extension period of control group; # *P* < 0.05, ## *P* < 0.01 VS the thawed 30 min or thawed 240 min period of NAC group

AV/PI reagents and flow cytometry were used to detect early apoptotic sperm, living sperm and dead sperm. AV/PI double positive cell indicates late apoptosis or necrotic spermatozoa, and AV^+^/PI^−^ cell indicates early apoptotic spermatozoa. As can be seen from the AV-PI staining scatter plot (Fig. 5a), the number of spermatozoa stained AV^+^/PI^−^ in after 30 min and 240 min of thawing was significantly increased than that at the extension period and cooling period, indicating that thawing could induce boar spermatozoa early apoptosis. Moreover, the number of early apoptotic cells in thawed spermatozoa were decreased in the NAC group compared with that in the control group (Fig. 5a) (*P < 0.05).

The relative expression of proteins in the spermatozoa of the extension period was expressed as 100%, and the changes in the expression of each apoptosis-related protein in the thawed spermatozoa were compared. Western blotting showed that the relative expression of Bcl-2 in thawed spermatozoa was significantly decreased compared with that in spermatozoa at the extension period and cooling period, and the relative expression of Bax, caspase-3, caspase-8, and caspase-9 was significantly increased in the control group. In the NAC group, the effect was reversed (Fig. 5b).

## Discussion

Sperm can be divided into high- and low- quality antifreeze sperm based on the quality of freeze-thawing, but the assessment methods of conventional sperm quality cannot be used to predict the antifreeze activity of fresh sperm before freezing [16, 28]. Therefore, researchers have been trying to identify anti-freeze markers. Usually, these marker proteins are membrane channel proteins or antiretroviral proteins that can respond to osmotic pressure and thermal pressure. The main proteins found to predict a sperm’s antifreezing ability are heat shock protein HSP 90A1 [29], parietal binding protein ACRBP, propyl phosphate isomerase TPI [30], and voltage-dependent anion channel protein VDAC2 [31]. However, these markers are difficult to apply in production practices.

ROS are byproducts naturally produced by cells during normal metabolism and play an important role in cell signal transduction, maintenance of homeostasis and other biological processes. However, during sperm freezing, the antioxidase in the sperm faces denaturation and decomposition, and the sperm produces excess ROS, leading to oxidative stress [12, 16]. Under high concentrations of ROS interference, the mitochondrial membrane potential is changed, the number of mitochondria is reduced, mitochondrial DNA is damaged, and the ATP levels are significantly reduced to cause cell apoptosis in sperm [32–34]. This paper aimed to evaluate whether ROS could be used as a predictor of boar-sperm cryotolerance. Therefore, we investigated the effects of the different steps of cryopreservation (extension, cooling, and thawing for 30 min and 240 min) with or without boar-sperm antioxidant (NAC) in boar sperm.

The ROS level was higher during the thawed period than during the extension and cooling periods and was more significant after 240 min than after 30 min of thawing. However, NAC added to the extender decreased the ROS level during the thawed period. Sperm motility is an important condition to ensure that sperm pass through the female reproductive tract, meet with the egg and penetrate the oocyte and is an effective indicator of semen quality. Our results showed that motility markedly declined during the thawed period with increased thawing time, and the addition of NAC increased the motility of the thawed period. Compared with other mammalian sperm, pig sperm are more sensitive to this oxidative stress reaction because the porcine sperm plasmid contains a higher proportion of polyunsaturated fatty acids that undergo lipid peroxidation with excess ROS [21]. The plasma membrane integrity of the thawed boar sperm was decreased compared with that during the extension and cooling periods and was increased with the addition of NAC. ROS can also damage the mitochondrial membrane, change the potential difference in the mitochondrial membrane, change the respiratory function of mitochondria, reduce ATP produced by mitochondria, and decrease the motility of sperm after thawing [35, 36]. Transmission electron microscopy showed that, in addition to the abnormal size, morphology and karyotype of thawed sperm, the ultrastructure of mitochondria exhibited pathological changes, the number and distribution of mitochondria were abnormal, the mitochondrial membrane potential decreased, and the level of ATP decreased; these effects were improved with the addition of NAC. ROS can attack the sperm cell membrane and sperm mitochondria, resulting in dysfunction of the cell membrane. Cytochrome C is released from the mitochondria into the cytoplasm, where it binds to apoptotic protease activator 1 (Apaf-1), activates caspase-9, and then caspase-3, caspase-6 and caspase-8 proteolytic enzymes and initiates apoptotic processes in cells. Western blotting showed that the expression of caspase-3, caspase-8, caspase-9, and Bax was increased, Bcl-2 expression of was decreased in thawed sperm; the addition of NAC reversed the change.

## Conclusion

Freeze-thawing of boar spermatozoa impairs their motility, plasma membrane, mitochondrial function, DNA integrity and apoptosis as a result of excessive ROS production. However, the extent of this deterioration was reversed by the addition of the antioxidant NAC into the freezing extender. These results indicate that ROS levels may underlie sperm cryotolerance in boars.

## Methods

### Chemicals and extenders

Unless specified, all the chemicals were purchased from Beijing Solarbio Science & Technology Co. (Beijing, China). The basic extender used for sperm dilution was modified Modena solution (mMS). The freezing extender used for sperm cryopreservation was supplemented with 20% (v/v) aseptic fresh egg yolk, 0.25% (v/v) Equex STM™ (Nova Chemical sales, Scituate, MA, USA) and glycerol (100 mM as the final concentration − 1.5% (v/v)).

### Semen collection

The boars (uncastrated male pigs) used for the experiments were provided by Jiangsu Academy of Agricultural Sciences. Pigs were not sacrificed; semen was collected by the hand-holding method and diluted with mMS (1:4) before being transported to the laboratory. After collection, the boars continued to be raised. The semen samples were kept in a Styrofoam box at 37 °C, which was the body temperature of the boar and transported within 1 h. Using computer-assisted sperm analysis (CASA, Hamilton Orne, Inc., HTM-HELOS, Beverly, MA, USA), only semen samples with 80% or higher sperm motility and 70% or higher anterograde movements were used.

### Spermatozoa cryopreservation

Diluted semen samples were centrifuged (450×g, 5 min, room temperature), and the concentration was adjusted to 1 × 10^9^ cells/ml by suspension in a freezing extender (1:1) or freezing extender with N-acetyl L-Cysteine (NAC) (1:1), a ROS inhibitor, before cryopreservation. The sperm were slowly cooled to 4 °C for 3–4 h. Thereafter, the semen was loaded into 0.25-mL plastic straws and fumigated for 10 min at 3 cm from the liquid nitrogen surface. Next, the semen was immediately submerged in liquid nitrogen at − 196 °C for storage, and the frozen time of the semen was 12 weeks.

### Experimental design

After centrifugation, the samples were divided into the following 2 experimental groups: control group without any antioxidant additive and NAC group with 3 mM NAC added to the freezing extenders. The ROS, spermatozoa motility, plasma membrane integrity, mitochondrial membrane potential, mitochondrial ultrastructure, ATP content, DNA integrity, apoptosis, and apoptotic markers (Bcl-2, Bax, PCNA, caspase-3, caspase-8, and caspase-9) were evaluated for each group during the extension period, cooling period, and the thawing times of 30 min and 240 min.

### Thawing of frozen spermatozoa

The straws containing spermatozoa were thawed in water at 37 °C for 30 min and 240 min. The spermatozoa were diluted in prewarmed (37 °C) MS (1:2) and washed twice by centrifugation (450×g, 5 min, 37 °C). Each pellet was resuspended in 1 ml of MS and then was used for detection.

### Evaluation of spermatozoa motility

The percentage of sperm motility at each step of cryopreservation was determined using a computer-assisted semen analysis system (CASA, with the Sperm Motility Analysis System software, Digital Image Technology, Tokyo, Japan). For each sample, three subsamples were analysed, 2 μL of each subsample was placed on an objective micrometer (Fujihira Industry, Tokyo, Japan), and a minimum of 300 sperm per subsample was analysed.

### Plasma membrane integrity

The plasma membrane integrity of the sperm was assessed by the hypoosmotic swelling test (HOST) (Revell & Mrode, 1994). Briefly, 50 μL of thawed semen was incubated with 1 ml of hypoosmotic solution (comprising 7.35 g/L of sodium citrate and 13.51 g/L of fructose) at 37 °C for 30 min. Next, the percentage of sperm with coiled or swollen tails was estimated at 37 °C using a phase contrast microscope (Nikon) at 9, 400 magnification; at least 200 of the sperm were counted per sample.

### Mitochondrial membrane potential

In total, 1 × 10^6^ spermatozoa were washed twice with PBS and suspended in 0.5 mL of JC-1 (Invitrogen, Molecular Probes; 5, 5′, 6, 6′-tetrachloro-1, 1′, 3, 3′-tetraethyl benzimidazolyl-carbocyanine iodide) working fluid for 30 min at 37 °C in a 5% CO_2_ incubator. Next, the working fluid was centrifuged for 5 min (450×g; 5 min; room temperature), and spermatozoa were washed twice with PBS and suspended in 0.5 mL PBS. Thereafter, flow cytometry was performed. The mitochondria of healthy cells containing red JC-1 aggregates were detected using FL2 channels; apoptotic or unhealthy cells containing green JC-1 monomers were detected using FL1 (FITC) channels.

### Determination of the ROS levels

The levels of intracellular ROS were detected using the 2′,7′-dichlorodihydrofluorescein diacetate (DCFH-DA; Molecular Probes) assay. Briefly, semen at different stages was collected, washed twice with MS, incubated with 10 μM DCFH-DA in 10 mL of MS at 37 °C for 30 min, and protected from light. After incubation, the cells were washed with MS, harvested with trypsin/EDTA and evaluated by flow cytometry (FACSCalibur; Becton Dickinson). Ten thousand cells were measured for each experimental condition. Fluorescence was detected at an excitation wavelength of 485 nm and an emission wavelength of 530 nm using a fluorescence microplate reader. Three experiments were performed in triplicate for each experimental condition.

### Mitochondrial ultrastructure

The spermatozoa were washed twice with 10 mL of 0.85% NaCl and centrifuged (450×g, 5 min, 37 °C), and then the supernatant was discarded. Thereafter, the spermatozoa were slowly added with 2.5% glutaraldehyde stationary solution along the inner wall of the centrifugal tube, fixed for 2–4 h at 4 °C, fixed with osmium acid, washed with distilled water, embedded with Epon 812 epoxy resin, prepared into semi-thin sections, and treated with formaldehyde. Aniline blue staining was used for localization under a light microscope. Ultrathin sections with spermatozoa were collected and observed by transmission electron microscopy (JEM2400-EX).

### Sperm chromatin structure assay

After the semen was washed 3 times with PBS, the spermatozoa were collected by centrifugation (450×g; 5 min; 37 °C), adjusted to 2 × 10 [4]/mL, smeared, naturally dried, fixed for 5 min with anhydrous ethanol-glacial acetic acid (3:1), and then stained for 10 min by placing in a dye tank containing freshly prepared acridine orange dye. After cleaning, drying, and sealing with paraffin oil, sperm were observed and counted under a fluorescence microscope. DNA double-stranded sperm are green, indicating the ability to fertilize. The DNA double-stranded sperm appear red or yellow, indicating insemination. The experiment was performed by counting 300 sperm.

### Measurement of ATP

Spermatozoa were collected by centrifugation (450×g; 5 min; 37 °C), suspended in 300–500 μL of hot double steam water, and placed in a hot bath (90 °C–100 °C) for 10 min. Thereafter, the levels of ATP were measured by ELISA (S0026; Beyotime, China) according to the manufacturer’s instructions.

### Apoptosis assay

Spermatozoa apoptosis was assessed using the FITC-Annexin V/PI Apoptosis Detection kit (556,547; BD Pharmingen, USA) as previously described [37]. Spermatozoa were washed twice with PBS, suspended in 1× binding buffer, and stained with Annexin V-FITC and propidium iodide (PI) (5 μg/mL each) in the dark for 15 min. Flow cytometry detection was immediately performed, and 10,000 events were collected for each sample.

### Western blotting

Spermatozoa were collected by centrifugation (450×g; 5 min; 37 °C), and 1 × 10^6^ cells/mL were diluted in 500 μL of SDS lysis buffer containing 6% SDS, 1 mM benzamide, 1 mM Na_3_VO_4_, 50 μM NaF, 50 μM pyrophosphate, 1 mM PMSF and 125 mM Tris-HCl at pH 7.5, supplemented with “cOmplete, EDTA-free Protease Inhibitor Cocktail” (Roche, Indianapolis, IN). The lysate was centrifuged (700×g; 5 min; 4 °C), and the supernatant was collected for protein analysis. The proteins were resolved by SDS-PAGE (12% acrylamide) and transferred onto a polyvinylidene fluoride membrane (PVDF), which was blocked with 5% nonfat milk for 2 h and then incubated with primary antibodies (Santa Cruz, USA) at 4 °C overnight. Thereafter, the membranes were incubated with horseradish peroxidase-conjugated secondary antibodies. The signals were detected using enhanced chemiluminescence (ECL) detection reagent (Thermo Scientific, USA). The images were photographed using BioDoc It (UVP Co, USA), and the intensity was analysed using LabWorks 4.5 software.

### Statistical analysis

Prism software (GraphPad Software, CA, USA) was used to analyse the data. The data are expressed as the mean ± S.E.M. Statistical evaluation of the results was performed by ANOVA followed by Bonferroni’s test. The results were considered significant at *P* < 0.05 and *P* < 0.01.

## Supplementary Information

**Additional file 1.**

## Data Availability

The datasets used and/or analyzed during the current study are available from the corresponding author on reasonable request.

## Abbreviations

Apaf-1: Apoptotic protease activator 1
CASA: Computer-assisted sperm analysis
DCFH-DA: 2′, 7′-Dichlorodihydro-fluorescein diacetates
ECL: Chemiluminescence
NAC: Acetylcysteine
PCNA: Proliferating cell nuclear antigen
PVDF: Polyinylidene fluoride membrane
ROS: Reactive oxygen species
UFA: Unsaturated fatty acids
