# Supplementary material for: Freeze-thawing impairs the motility, plasma membrane integrity and mitochondria function of boar spermatozoa through generating excessive ROS
Source: BMC Vet Res. 2021 Mar 22;17:127. doi: 10.1186/s12917-021-02804-1 (PMC7986419; doi:10.1186/s12917-021-02804-1)
Supplement: Supplementary file 1 — Additional file 1. [file 12917_2021_2804_MOESM1_ESM.docx]

**All original, full-length gel and blot images for Figure 6B:**

**Samples:**

1-4: Control group (Extension period,

Cooling period,

Thawed 30 min,

Thawed 240 min)

5-8: NAC group (Extension period,

Cooling period,

Thawed 30 min,

Thawed 240 min)


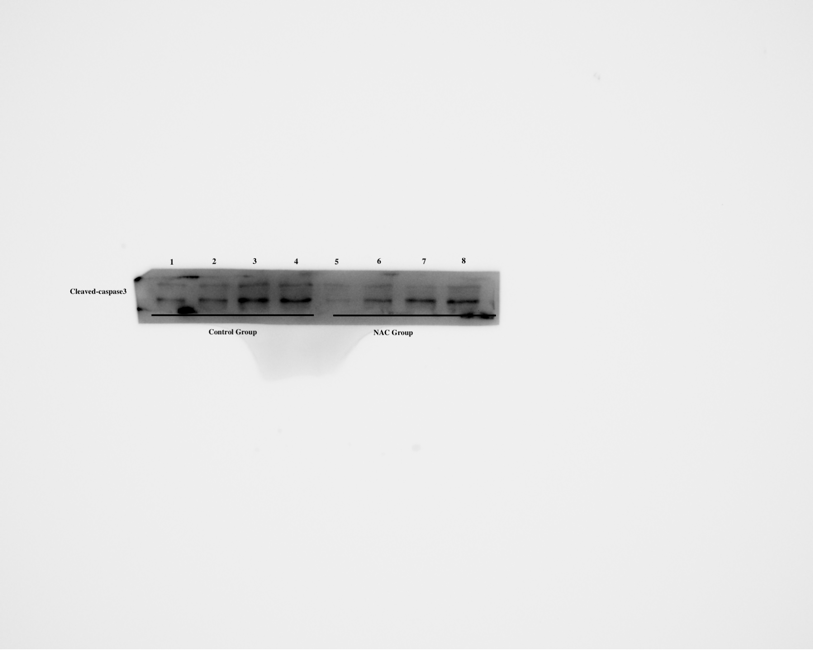


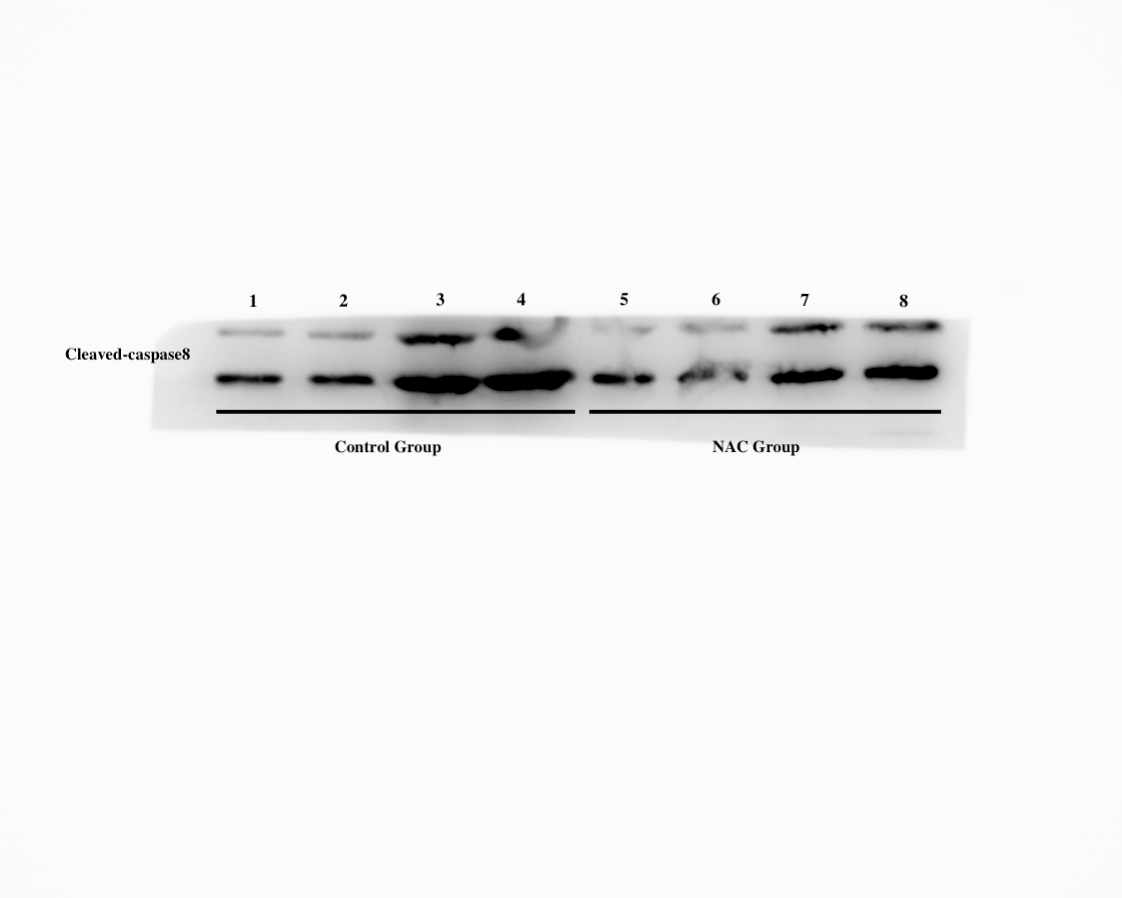


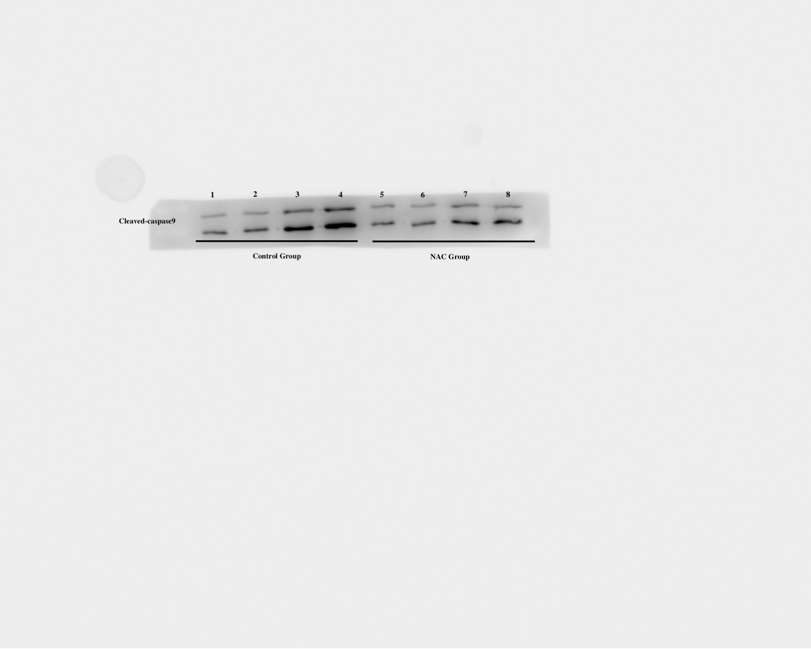


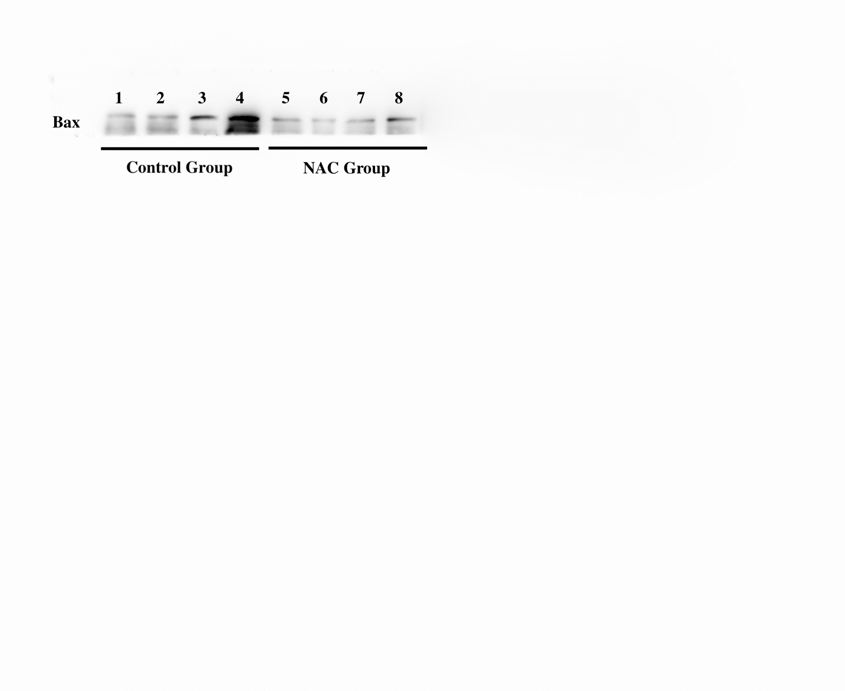


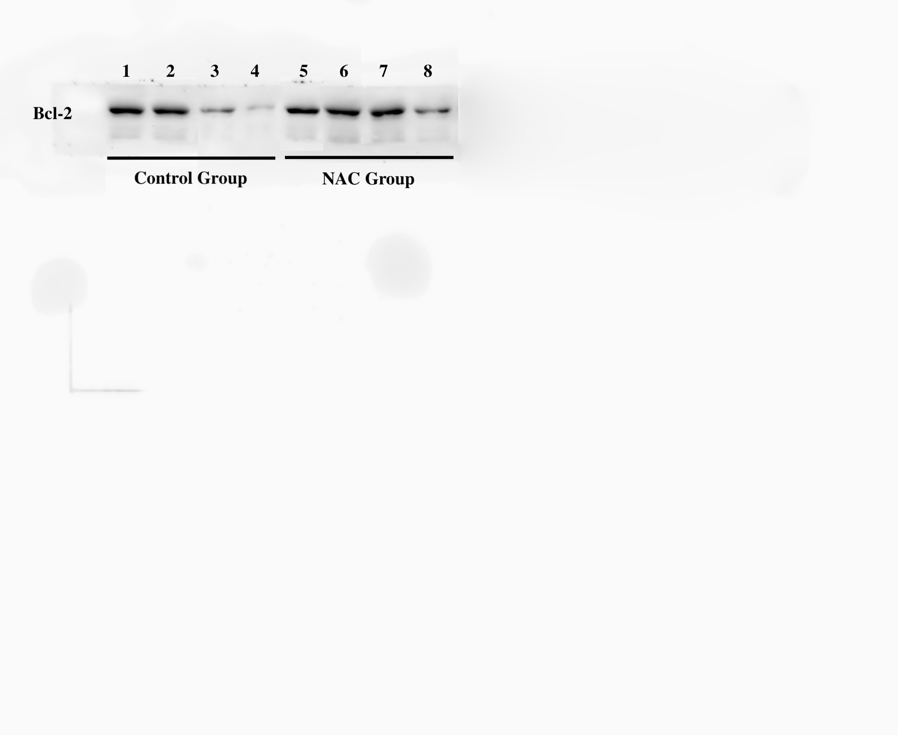


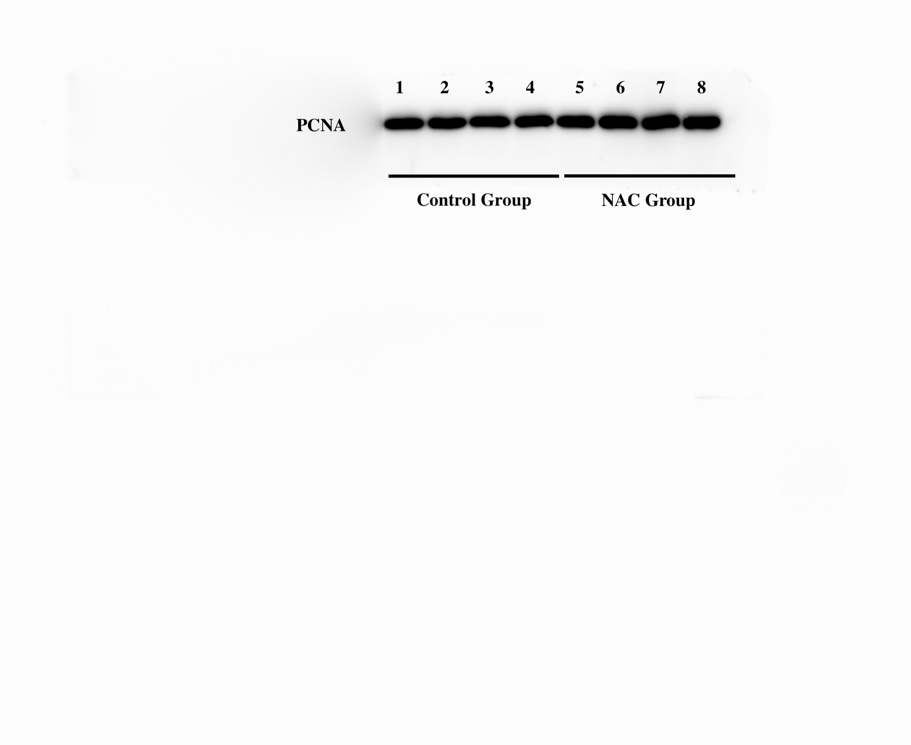


**Figure legends:** Apoptosis of sperm was detected by western blot (B).
